# Digital learning, face-to-face learning and climate change

**DOI:** 10.1016/j.fhj.2024.100156

**Published:** 2024-06-25

**Authors:** David Liam Davies, AbdulAzeez Lawal, Angela E. Orji, Chloe Tytherleigh, Kieran Walsh

**Affiliations:** BMA House, Tavistock Square, London WC1H 9JR UK

**Keywords:** Digital learning, Medical education, Climate change

## Abstract

Debates about digital learning, face-to-face learning and blended learning often focus on their effectiveness in achieving a few core educational outcomes. The cost or convenience of using different methods to achieve certain outcomes have increasingly come into the educational framework over the past two decades. However, only rarely do educators or learners consider the climate footprint of their various activities. This is an important shortcoming, as all learning activities can contribute to our overall climate footprint. Providers of education should do their best to minimise the carbon footprint associated with their learning. But learners also have responsibility to ensure that how they access learning is also associated with minimal environmental cost. Both providers and learners should focus on activities that are likely to have the greatest impact. This is relevant both to face-to-face education and digital learning.

Debates about digital learning, face-to-face learning, and blended learning often focus on their effectiveness in achieving a few core educational outcomes. These include applied knowledge, problem-solving or procedural skills, and professional behaviours. The cost or convenience of using different methods to achieve certain outcomes have increasingly come into the educational framework over the past two decades.^1^ This is important as it enables learners and providers of education to access and deliver education that is ideally high quality for the lowest possible cost. However, only rarely do educators or learners consider the climate footprint of their various activities.^2^ This is an important shortcoming, as all learning activities can contribute to our overall climate footprint. The ongoing climate crisis means that we should be doing all that we can – however large or small – to reduce that footprint, especially given the wider global healthcare implications. More extreme weather, increasingly poorer air quality, and threats to food security are just a few problems that affect patients. In the following perspective paper, we will outline a number of considerations that should be taken into account when linking together learning activities, learning outcomes, and carbon footprint.

Face-to-face learning is the traditional means of delivering medical education activities, and it has much to commend it. Students and trainees can learn knowledge, skills and behaviours in face-to-face settings and also have the opportunity to network and engage in informal learning with educators and peers. The face-to-face environment can especially help with learning practical or procedural skills and professional behaviours. However, travel is often required for face-to-face learning.^3^ This can involve driving and/or using public transport, which can include air travel to events such as international conferences. Travel to some in-person conferences can produce around 1,000 times more metric tonnes of CO_2_ than a virtual conference, with over 90% of these emissions potentially being attributed to travel.^4,^^5^ It is possible for delegates or organisers to calculate the carbon footprint of different components of face-to-face learning; however, Leddin *et al* suggest that providers should us a simplified model to do this 'using flying distance only, to estimate travel-related emissions'.^6^ Using public transport is the most climate-friendly means of travelling. Hotel accommodation is also required if people are traveling for a meeting that lasts longer than a day. This will have its own environmental effects – such as the use of detergents for laundry of beddings and towels. Physical events are often associated with usage of implements such as single-use plastics and polystyrene food packages. These are not a necessity, but they are a common accompaniment of face-to-face learning events. This is not a comprehensive account of the carbon footprint of face-to-face learning, but it should encapsulate most of the activities that can contribute – namely travel, accommodation and subsistence.^7^

Digital learning can also enable multiple useful learning outcomes. It can enable learning knowledge and the learning of certain skills and behaviours. Technological advances mean that certain learning outcomes that until recently would have only been possible with face-to-face learning (such as communication skills training and improvement) are now quite feasible with e-learning. And digital learning is usually more convenient than having to travel. Digital learning also has advantages in terms of its carbon footprint over face-to-face learning. Digital learning does have a carbon footprint, but it is generally less than the carbon footprint involved in travel.^8^ The carbon footprint associated with digital learning involves electricity, hardware and software – to create and access learning materials. Electronic devices have varying degrees of climate-friendliness. Some devices consume more electricity than others – generally, desktop computers set up for advanced gaming or simulation activities consume the most.

So how best to balance all of this? There is a clear opportunity to get the best from digital and face-to-face learning and to minimise carbon footprint at the same time.

It is possible to reduce the amount of face-to-face learning that is required to achieve needed learning outcomes and to replace unnecessary face-to-face learning with digital learning. One example is that lectures can be recorded or delivered live online. If travel is necessary, flying should be avoided apart from when there is no alternative. Public transport should be the preferred means of travel. Physical events providers should take all means possible to reduce their carbon footprint, from avoiding single-use plastics to providing environmentally friendly meals and staying in accommodation close to the conference.

If digital learning is to be employed, learners should utilise devices that are the most environmentally friendly. These might be devices that satisfy energy efficiency standards and that use less power. It might also be preferable to use devices with a longer life or constructed with environmentally friendly material or that can be recycled. Another consideration is the use of artificial intelligence in digital learning. Many artificial intelligence (AI) programmes are based on machine learning, and this can have a significant environmental impact.^9^ This is because of the energy requirement associated with this form of computing. There are ways to reduce this carbon footprint, such as by reducing the computational demands of machine learning, using renewable energy sources and/or thinking through the lifecycle of machine learning programmes from deployment to maintenance and eventually to replacement. AI programmes can enable more personalised learning, but it is a matter of balancing this against increased carbon footprint. There is some evidence that younger doctors have a greater preference for e-learning than older generations, but there is not much evidence that concerns about climate change are affecting their preferences.^10^

In summary, it is clear that there are no right or wrong answers as to how to provide effective learning with minimal environmental cost. Providers of education should do their best to minimise the carbon footprint associated with their learning. But learners also have responsibility to ensure that how they access learning is also associated with minimal environmental cost. Both providers and learners should focus on activities that are likely to have the greatest impact. While avoiding unnecessary flights to international conferences will likely make a much larger difference, the cumulative effect of eliminating single-use plastics at face-to-face meetings cannot be overstated. And of course, both providers and learners share a responsibility that they get the most out of any form of learning that they take part in. Sometimes this might mean blending face-to-face and digital learning – there is some evidence that this may help with the transfer of learning to practice.^11^ It would also be good practice for providers of all forms of learning to publish the carbon footprint of their programmes and efforts that they are making or have made to reduce this.

## Declaration of competing interest

KW works for BMJ which produces digital learning and clinical decision support resources such as BMJ Learning and BMJ Best Practice.
